# Reproductive health challenges during a flood: A qualitative study

**DOI:** 10.1002/nop2.2044

**Published:** 2023-11-29

**Authors:** Farzaneh Safajou, Fatemeh Nahidi, Fazlollah Ahmadi

**Affiliations:** ^1^ Student Research Committee, School of Nursing and Midwifery Shahid Beheshti University of Medical Sciences Tehran Iran; ^2^ Midwifery and Reproductive Health Research Center, Department of Midwifery & Reproductive Health, School of Nursing & Midwifery Shahid Beheshti University of Medical Sciences Tehran Iran; ^3^ Nursing Department, Faculty of Medical Sciences Tarbiat Modares University Tehran Iran

**Keywords:** floods, Iran, natural disaster, qualitative research, reproductive health

## Abstract

**Aim:**

The study aimed to investigate women's reproductive health challenges during floods.

**Design:**

This study is qualitative, specifically employing content analysis with an inductive approach.

**Methods:**

Data were collected through in‐depth, semi‐structured individual interviews between July and December 2021. The study involved 13 women affected by floods in Golestan province, Aq Qala Township, and also included seven healthcare providers and officials. Before the interviews, informed and written consent was obtained from all participants. The sampling process continued until data saturation was achieved.

**Results:**

The analysis of the participants' experiences in this study revealed four main categories of requirements, which were as follows: Maternal and Child Health with four subcategories, Essentials of Women's Health Care with two subcategories, Problems of Relationships with two subcategories, and Aggression and Physical Violence with two subcategories. In conclusion, during floods, women encounter numerous challenges in preserving their reproductive health. Recognizing and understanding these challenges can be instrumental in effectively planning measures to prevent or address them during disasters like floods.

**Implications for the profession and/or patient care impact:**

Every disaster has unique conditions and challenges. The health requirements of individuals impacted by floods differ from those affected by other natural disasters. By identifying the specific reproductive health needs of women affected by floods, midwives and other healthcare providers can enhance their planning efforts, enabling them to better address and fulfil these needs during such critical situations.

**Patient or public contribution:**

Thirteen women were affected by floods, and seven healthcare providers and officials were interviewed.

## 1 INTRODUCTION

Floods are considered the most destructive natural disaster, impacting human lives worldwide (UNISDR, 2015). Significant floods occur yearly in Iran, mainly due to the country's extensive area, diverse climates, and variable precipitation patterns in various catchment areas, particularly in the northern regions (Ardalan et al., 2009).

Floods can result in various adverse psychological and physiological consequences, particularly concerning reproductive health (Mallett & Etzel Ra Md, 2018). Additionally, access to reproductive health services is often constrained during disasters (Nakhaei et al., 2015).

## 2 BACKGROUND

Disregarding the needs of sexual and reproductive health (SRH) in critical environments can lead to severe consequences, many of which can be mitigated or prevented through appropriate planning. These consequences include maternal and infant mortality and morbidity in terms of physical and mental health. Furthermore, there are ramifications of unwanted pregnancies, such as unsafe abortions and the implications of sexual violence, which may result in unwanted pregnancies, increased sexually transmitted infections (STIs), heightened HIV transmission, and mental health issues, including depression and trauma (Frankenberg et al., 2014).

The findings of a study conducted in Iran revealed that, following an earthquake, reproductive health needs, such as the availability of sanitary pads and meeting the requirements of pregnant women, were not adequately addressed. Moreover, the lack of proper health conditions, limited healthcare services, and insufficient privacy in healthcare facilities hindered the provision of reproductive health services. Reproductive health services, including prenatal care, obstetrics, and emergency obstetric care, were rarely accessible (Nakhaei et al., 2015).

In response to these health needs, the Interagency Reproductive Health Contingency Working Group (IAWG) has developed the Interagency Reproductive Health Contingency Field Manual (IAFM). One of its crucial components is the Minimum Initial Service Package (MISP) for Reproductive Health in Unforeseen Incidents. The MISP consists of a set of reproductive health services and equipment to establish minimal conditions in a disaster. These can be implemented without prior assessment in any crisis environment, whether it is a human‐made or natural disaster (Foster et al., 2017; Krause et al., 2011). Once the situation stabilizes and preparations for comprehensive SRH services are underway, it becomes essential to plan for obtaining input from the affected community regarding the initial response. This step helps identify gaps, successes, and areas for improvement. The MISP serves as the initial building block for SRH programming in crises (Interagency Working Group, 2018).

Sohrabizadeh et al. (2018) conducted a study to identify reproductive health management challenges during disasters in Iran. The study highlighted the importance of developing national and local plans within the health system to effectively meet the reproductive health needs of people during such crises. These plans should prioritize accessing essential SRH care information and services without discrimination. By incorporating these aspects into disaster management plans, the health system can better address the reproductive health requirements of individuals affected by disasters (Sohrabizadeh et al., 2018).

An effective and efficient response to the reproductive health needs of women affected by disasters, such as floods, relies on providing services tailored to their specific requirements, conditions, and cultural and geographical context and having well‐defined written plans and standard instructions that align with the cultural and geographical aspects. It is essential to gather appropriate data to plan and evaluate interventions and actions that can be lifesaving in critical situations (Pyone et al., 2015). In Iran, few studies have investigated women's needs after floods. Given that cultural, political, and economic conditions influence women's roles and needs, it becomes necessary to comprehend women's experiences within each distinct culture (Nakhaei et al., 2015). Consequently, any study to address this issue should start with qualitative research to comprehensively understand the reproductive health needs in such incidents.

The insights obtained from this research can assist researchers and healthcare professionals in understanding women's reproductive health requirements during floods. Consequently, this knowledge can lead to the formulation of more effective measures for providing appropriate care for women in flood‐affected areas. Thus, this study aimed to identify and assess the reproductive health challenges flood‐affected women face.

## 3 METHODS

This study adopts a qualitative approach, specifically utilizing content analysis with an inductive method.

### 3.1 Data collection

Data collection for this study involved conducting in‐depth, semi‐structured interviews between July and December 2021. Before the interviews, the participants were provided with a clear and concise explanation of the study's objectives. The interview commenced with one or two general questions, such as asking the participants to describe their experience on the day of the flood incident or inquire about specific challenges they faced as women during the flood. Subsequently, detailed questions were asked to gather comprehensive information. Each interview lasted 30–90 min, with an average duration of 45 min. The data were recorded using a tape recorder, and the interviewees' emotions, facial expressions, and body language were also carefully noted during the interviews.

### 3.2 Participants

In this study, participants, including healthcare providers and flood‐affected women with substantial experience who could share their insights, were purposefully selected from healthcare centres in flood‐affected areas of Golestan province, including Aq Qala Township and surrounding villages. The data collection took place approximately 2 years after the flood.

Regarding the inclusion criteria, the study considered flood‐affected women within the childbearing age range of 15–49 years. Additionally, female healthcare providers and reproductive health professionals were eligible to participate. In qualitative studies, there is no specific criterion for sample size. In this study, the sampling process continued until data saturation was achieved, meaning new information or insights were no longer emerging from the interviews.

### 3.3 Data analysis

The data collected from the interviews were subjected to thematic analysis using the Granheim method. Immediately after recording each interview, it was transcribed verbatim. The analysis of each interview was carried out promptly, enabling the insights obtained to inform the data collection process in subsequent interviews.

Specifically, each interview was read multiple times to gain a comprehensive understanding of its meaning. Codes were extracted and assigned specific names through condensing and abstracting procedures. Codes that exhibited similarities were grouped as subcategories. In the next step, these subcategories were further categorized into broader categories based on their content similarities. This systematic process allowed for rigorous data analysis, facilitating the identification of significant themes related to the reproductive health challenges flood‐affected women face.

### 3.4 Rigour

Credibility, dependability, confirmability, and transferability were carefully examined to ensure the study's trustworthiness (Lincoln et al., 1985). Several measures were taken to meet these criteria: In‐depth and lengthy interviews were conducted, allowing for a thorough exploration of the participants' perspectives. Repeated questions were utilized during the interviews to minimize any ambiguities in the questions and ensure accurate responses. Participants were also allowed to review the transcriptions of their interviews to verify the accuracy of the data. Furthermore, supervisors reviewed the study findings to confirm that the identified categories aligned with the participants' statements. In addition, the data analysis process was shared with two external experts familiar with qualitative research methods. Their input and agreement on the identified categories and subcategories added objectivity and validation to the findings.

### 3.5 Ethical considerations

#### 3.5.1 Redacted

The Ethics Committee approved the study protocol (IR.SBMU.PHARMACY.REC.1399.202). The study's objectives and methods were clearly and comprehensively explained to all participants. Informed and written consent was obtained from both flood‐affected women and other participants, signifying their voluntary agreement to be included in the study and having their interviews recorded. Moreover, all participants were assured that their information would be treated with strict confidentiality to protect their privacy and identity.

## 4 FINDINGS

In this study, a total of 13 flood‐affected women, seven healthcare providers, and relevant officials were interviewed. Tables 1 and 2 present the participants' demographic information. From thoroughly analysing the participants' experiences, four distinct categories emerged: Maternal and Child Health, Essentials of Women's Health Care, Relationship Problems, and Aggression and Physical Violence (Table 3).

**TABLE 1:** Characteristics of flood‐ridden women.

Number of participants	Age	Marital status	Education	Job status	Fertility status
1	32	Married	High school diploma	NO	Pregnant
2	32	Married	Bachelor	NO	Using contraceptives
5	25	Married	High school	NO	Pregnant
6	28	Married	Bachelor	Tailoring	Pregnant
9	34	Married	High school diploma	NO	Using contraceptives
10	37	Married	High school diploma	NO	Breastfeeding
11	25	Married	High school diploma	NO	Breastfeeding
14	26	Married	High school diploma	NO	Other
15	24	Married	High school diploma	NO	Breastfeeding
16	33	Married	High school diploma	NO	Using contraceptives
17	22	Married	High school diploma	NO	Pregnant
18	18	Married	High school	NO	Pregnant
19	21	Married	High school diploma	NO	Using contraceptives

**TABLE 2:** Characteristics of health care providers as well as officials.

Number of participants	Age	Marital status	Education	Job status
3	34	Married	Bachelor	Midwife
4	39	Married	Bachelor	Midwife
7	30	Married	Bachelor	Health worker
8	28	Single	Bachelor	Health worker
12	36	Married	Master	Head of Education and Health Promotion Unit
13	31	Married	Bachelor	Midwife
20	50	Married	Bachelor	Head of health and family planning and nutrition unit

**TABLE 3:** Formation of the concept of reproductive health challenges during floods.

Subcategory	Category	Theme
Prenatal and postpartum care	Maternal and Child Health	Reproductive health challenges during floods
Nutrition of pregnant and lactating mothers		
Child nutrition		
Distribution of contraceptives		
Attention to menstrual health	Essentials of Women's Health Care	
The needs of high‐risk and vulnerable women.		
Problems of sexual relationships	Problems of Relationships	
The problem of unhelpful spouses		
The feeling of insecurity of women and girls against aggression	Aggression and Physical Violence	
Physical violence against pregnant women		

### 4.1 Maternal and child health

This category encompasses four subcategories, which are as follows: prenatal and postpartum care, nutrition of pregnant and lactating mothers, child nutrition, and distribution of contraceptives.

#### 4.1.1 Prenatal and postpartum care

Pregnant women are particularly vulnerable during floods, requiring special attention from the midwifery team. One of the primary steps taken by the team is to locate and monitor the status of pregnant women, particularly those identified as high‐risk cases. This proactive approach is crucial to ensuring the well‐being and safety of pregnant women during such critical situations.

I had a list of pregnant women, and I approached each, asking questions like: Where are you located? How are you feeling? What are your current health conditions? I advised them to undergo certain tests. Subsequently, we followed up by calling them again to check on the progress of their tests and ultrasound scans. (P. 3, healthcare provider)

In addition to the measures mentioned earlier, efforts were made to distribute pregnancy supplements, offer free tests, and provide ultrasound services to pregnant women. These initiatives ensured pregnant women received support and care during the floods. In emergencies, pregnant women were promptly referred to the city hospital by ambulance to address any urgent medical needs they might have. These actions were taken to safeguard the health and well‐being of pregnant women in the face of the disaster. “We ensured proper coordination with the Emergency Medical Services (EMS) (115), as it was essential to promptly dispatch pregnant mothers to the hospital if the need arose.” (P. 20, health care provider).

According to the participants' experiences, postpartum mothers were found to be in unfavourable health conditions due to the challenging health environment caused by the floods. Additionally, there is a need to pay careful attention to the breastfeeding status of mothers during such critical situations. These findings highlight the importance of providing appropriate care and support to postpartum mothers in flood‐affected areas to ensure their well‐being and the health of their infants.

A mother who had undergone a cesarean section was subsequently admitted to a contaminated camp. The conditions in the camp were very unsanitary, with dirty tents, poor ventilation, and inadequate heating and cooling facilities. There was significant environmental pollution. The situation was very bad (P. 12, health care provider)

Breastfeeding was difficult. One of the mothers who had recently given birth during the flood was experiencing mental health issues. This mother faced difficulties with lactation, as she had insufficient milk production and lacked the motivation to breastfeed her infant (P. 13, health care provider)

#### 4.1.2 Nutrition of pregnant and lactating mothers

Pregnant and lactating women have specific nutritional requirements to ensure their well‐being during these critical periods. The participants emphasized the importance of addressing these nutritional needs, including avoiding repetitive daily meals and a lack of snacks. They also noted that it was essential to include all food groups, emphasizing consuming adequate milk, dairy products, and fruits and vegetables.

The food provided was satisfactory during the first two days. However, the food was served cold during lunch, which resulted in many people not eating it. Additionally, the meal schedule consisted only of breakfast, lunch, and dinner, with no snacks or other meals in between (P. 18, flood victim)

The food provided did not follow a balanced food pyramid. Instead, it predominantly consisted of rice, bread, and cheese. Dairy products were scarce, and there was a significant lack of vegetables and fruits. Fruits were entirely unavailable. Essential foods were unavailable, particularly for pregnant women (P. 8, health care provider)

Mothers experiencing nausea, vomiting, and pregnancy cravings (viar) required special nutritional attention during the flood period, as one of the healthcare providers states:

Mothers in the flood‐affected area were expressing concerns about their nutritional challenges. One mother, in particular, was suffering from viar and experiencing severe nausea, which made it difficult for her to eat anything. She expressed her distress in a sad and tearful voice, stating that the available foods were making her sick. Despite the awareness of her condition, I could not provide any suitable options to address the specific needs of this mother (P. 4, health care provider)

Breastfeeding mothers also require special attention to their nutritional needs, similar to pregnant women. However, their nutritional requirements were not adequately met during the flood period. “During the flood period, we could not drink enough tea or eat anything to increase our milk. They only served the main food. There were no fruits and such things” (P. 11, flood victim). And one of the healthcare providers also states, “The breastfeeding mother was stressed and stated: My breastmilk is not sufficient, what should I do? My nutrition is inappropriate; my breastmilk is not enough for the baby” (P. 13, healthcare provider).

#### 4.1.3 Child nutrition

Providing the necessary food for infants and children became a major concern for mothers and officials during the flood. One mother specifically highlighted the need for infant formulas to feed her child. However, the lack of proper equipment to boil water posed difficulties in preparing the formula safely. “I needed infant formula, they gave me one or two. We were upstairs, and went downstairs to get boiling water. It was hard for me to go down or up frequently” (P. 6, flood victim).

“A mother had given birth to twins and required infant formula and boiling water to feed them. She had to be given a flask to hold the boiling water. However, she had a bottle in her hand and was looking for boiling water” (P. 1, flood victim). The unscheduled distribution of baby formula during the flood posed a risk of baby formula misuse. “Due to the large quantities of baby formula distributed during the flood, there was an unfortunate increase in the number of formula‐fed babies. Some mothers stopped breastfeeding and shifted to using baby formula. However, these distributions were not adequately planned as no supervisor controlled baby formula distribution” (p. 12, healthcare provider).

Another crucial nutritional need during the flood was to pay attention to the diet of children under the age of one who were receiving complementary foods alongside breast milk. “We had a real problem for small children. After all, their complementary soup or special food was unavailable” (P. 20, healthcare provider). In addition to the main meals, children also require snacks to meet their nutritional needs adequately. However, providing snacks during the flood posed difficulties for mothers due to the challenging conditions. “The little children were upset. They said: “We want biscuits.” They wanted snacks. Where were the snacks? How can we give them snacks?” (P. 4, healthcare provider).

Paying attention to essential supplements for children is crucial, particularly when there is a risk of inadequate child nutrition. In this study, it was only considered in certain camps, while it was lacking in others, as one of the mothers says, “There were no vitamins. They did not give them to us” (P. 11, flood victim). On the other hand, one of the healthcare providers mentioned their observation about the distribution of supplements in some camps during the flood. “The only thing we did in the first days was to distribute supplements, including zinc syrup. We also administered daily multivitamins to children. Iron tablets were also distributed” (P.20, healthcare provider).

#### 4.1.4 Distribution of contraceptives

During the flood, contraceptives needed to be distributed to support family planning and reproductive health. However, according to the participants' accounts, contraceptives were not provided in the early days of the flood. Instead, they were only supplied when specifically requested by women after some time had passed. “We had no prior experience with floods, and we realized what we needed step by step. I can boldly say that the planning process began three to four days after the onset of the flood. That is to say, nothing had been planned from the first day.” (P. 20, healthcare provider).

One breastfeeding mother expressed the need for oral contraceptives specifically designed for breastfeeding women as follows: “I went to get breastfeeding pills several times, and each time, they asked if I was sexually active and why I wanted the pills. It felt like a personal invasion. Nevertheless, after 15 days, when I finally returned home, I had to start taking the pills.” (P. 11, flood victim).

These limitations and lack of access to family planning tools, especially in the early days of the disaster, also led to unwanted pregnancies. “We also had an unplanned pregnancy. One woman was taking low‐dose combined pills (LDs) but had forgotten to take them, and one or two other people also became pregnant at that time. Additionally, when we needed contraception, there were no tools available.” (P. 8, healthcare provider).

### 4.2 Essentials of women's healthcare

In addition to pregnant and lactating women, other women of childbearing age faced challenges maintaining their reproductive health during the flood. This category encompasses two subcategories: attention to menstrual health and the needs of high‐risk and vulnerable women.

#### 4.2.1 Attention to menstrual health

One of the critical issues related to menstrual hygiene during floods was the availability and distribution of sanitary napkins among women. “I was menstruating when the flood started, and there were no sanitary napkins available initially. However, they were brought gradually over time.” (P.9, flood victim).

The participants also highlighted the need for painkillers to relieve dysmenorrhea (menstrual cramps) and iron pills, particularly for menstruating adolescents: “My aunt had a teenage daughter who was menstruating at the time. It was likely only her third or fourth period, and she was experiencing pain and had a pale complexion due to poor nutrition. They needed painkillers and iron pills, but these were unavailable.” (P. 8, health care provider).

Some of the participants highlighted the challenges faced by menstruating girls living in camps, particularly due to feelings of embarrassment and the fear of experiencing menstrual bleeding that could result in bloody clothes: “Several teenage girls, around 16–17 years old, came to me and said they had started menstruating earlier than expected and had bled through their clothes. They did not know what to do.” (P. 12, health care provider). As reported by the participants, the delayed replacement of sanitary pads due to unsuitable conditions was another significant factor threatening menstrual health. “The young girl felt embarrassed about changing her pad inside the tent because her shadow would have been cast on the wall. We advised her to change it to avoid infection, but she remained embarrassed.” (P. 8, health care provider).

#### 4.2.2 The needs of high‐risk and vulnerable women

Drug‐abusing women are among the most high‐risk and vulnerable female groups, especially in critical situations such as floods. In this regard, one of the participants said, “In the early days, a specialist visited those addicted and prescribed methadone to prevent them from experiencing problems in the camps” (P. 12, health care provider). One of the healthcare providers talked about the women who were secretly abusing drugs without informing their families or relatives and who were not having their needs addressed: “Meanwhile, there were also women, secretly abusing drugs, whose problems no one knew about. They had difficulty obtaining their drugs and may have used acetaminophen codeine instead, possibly because they felt ashamed to seek methadone.” (P. 12, health care provider).

Women living with HIV and AIDS are also at high risk, and according to the participants, the secrecy and taboo surrounding the issue made it difficult to identify these women, and they were, therefore, likely to be overlooked. “We had no proper statistics, at least not that we knew of. HIV and AIDS are taboo topics, and their information is often kept secret. The Lean System did not provide any statistics, and even if they existed, they were likely ignored in the camps”. (P. 20, health care provider). On the other hand, the lack of awareness and taboos surrounding HIV in society led to the expulsion of a woman suspected of having the disease from the camping area.

There was a woman who worked as a garbage collector and was also addicted to drugs. Later, I discovered that she was the same woman whose tent had been taken down by others out of fear that she had AIDS. They had moved her tent to another location to burn it. After the flood had subsided, I spoke to her on the street, and she explained that people had thought she was sick and thrown her out. However, she was not HIV‐positive but was still at a high risk of contracting the disease. The people in nearby tents felt the risk and reported it to the authorities, who then took down her tent. (P. 12, health care provider)

Women and girls with physical disabilities were also vulnerable and needed special attention during the flood. “There was a woman with a disability who had left her cane in the house. Due to her loose legs, she had fallen twice or thrice. I coordinated with the Welfare Organization to get her a cane” (P. 13, healthcare provider).

### 4.3 Problems of relationships

This category consists of two subcategories: Problems of sexual relationships and the problem of unhelpful spouses.

#### 4.3.1 Problems of sexual relationships

During the flood, sexual relationships faced challenges due to a lack of a suitable place for intimate affairs. Moreover, the separation from spouses due to the flood could have further complicated sexual relationships, as reported by a participant: “There was no suitable place for us to have a sexual relationship because I was living with my parents. I was unable to have a private relationship with my husband.” (P. 14, flood victim). “We did not have a relationship at that time, since I only saw my husband one month later. There was no need for contraceptives. The conditions were not good” (P.15, flood victim).

Having no sex for a long time also caused tensions between couples and negatively affected people's moods. “The combination of the challenges posed by the flood and the difficulties of maintaining a sexual relationship during that time could cause a great deal of stress and frustration, leading to a loss of temper.” (P. 3, healthcare provider). Ablution after sexual intercourse is a religious obligation for Muslims. Some participants in the study mentioned that the lack of a suitable place for ablution during the flood was a reason for refraining from sexual activities.

Some people in tented areas had sexual relationships because they felt they had no other option. However, they faced difficulties performing ablution as there was no suitable place. This lack of a proper place for ablution caused significant distress and frustration for these individuals. (P. 4, healthcare provider)

#### 4.3.2 The problem of unhelpful spouses

During the flood, the separation of most men from their wives and families was common for various reasons. This situation resulted in women being left to manage most of the work and responsibilities alone, which created significant challenges for them. Some women talked about the difficulty of this situation: “I was alone during the flood because my husband struggled with addiction and could not help with the housework. Despite being eight months pregnant, I managed to tidy up our home alone. However, the furniture was completely soaked, making it difficult for me to move it.” (P. 5, flood victim).

The difficulty of caring for children alone was another problem mentioned by mothers. “If this happens again, for God's sake, treat women better. Men can manage independently, but women need more support during these times. They are often left alone to care for their children, with one child in their arms and another by their side. It is challenging for them, and men do not always take on the responsibility of caring for their children. As a result, the burden falls on the wives.” (P. 6, flood victim).

### 4.4 Aggression and physical violence

Due to the changing environmental conditions during floods, ensuring the safety of women and girls becomes increasingly challenging and of utmost importance. This category includes two subcategories: the insecurity of women and girls against aggression and physical violence against pregnant women.

#### 4.4.1 The feeling of insecurity of women and girls against aggression

The participants in the study expressed concerns about the low security of the camps during the flood. One of the reasons cited for this lack of security was the presence of strangers living in the camp, which led to a lack of familiarity among the residents. The presence of unfamiliar individuals in the camp created a sense of insecurity for women and girls, particularly when they needed to go to the bathroom or venture to places further away from their tents. “The living conditions in the accommodation were unsafe. One of my female cousins was accosted by a boy when she went to use the bathroom. The boy grabbed her by the collar and pulled her hair, causing her to scream in fear. Her mother saved her while she was very scared”. (P. 16, flood victim).

In some camps, rumours of a liaison fuelled these concerns. One of the participants said such rumours led her to hesitate to go to the settlements or communal areas: “I had heard some disturbing things about the atmosphere in the area. My brothers, who are very protective and jealous, warned me not to go there due to the unethical behavior that reportedly occurred there.” (P. 5, flood victim).

#### 4.4.2 Physical violence against pregnant women

In the study, some participants reported physical violence against pregnant women. One of the healthcare providers shared an account of a man physically assaulting his pregnant partner. “There was another woman in the camp whose husband was addicted to methamphetamine. He had physically assaulted his pregnant wife in front of others. This incident was deeply distressing for the woman that she might have considered suicide.” (P. 4, healthcare provider).

## 5 DISCUSSION

The study highlighted various challenges women face regarding reproductive health during floods, and one significant challenge identified was Maternal and Child Health needs. Access to health services becomes limited during floods, making it crucial to pay special attention to pregnant women and provide adequate care during pregnancy while meeting their needs.

The midwifery team played a crucial role in addressing the reproductive health needs of pregnant women during the flood. One of the first steps they took was to find and monitor the status of pregnant women, especially those at high risk. This proactive approach allowed the midwifery team to perform routine pregnancy care and necessary tests or ultrasonography to ensure the well‐being of pregnant women.

Prior studies have emphasized the importance of coordination among midwives and other healthcare providers to deliver essential reproductive health and maternal and newborn services in disaster‐affected areas. Collaborative efforts can ensure that contraceptive care, prenatal and postpartum care, access to laboratory tests, ultrasound imaging, and clean birth kits are available to women in need during floods (Hays & Prepas, 2015; Monteblanco & Leyser‐Whalen, 2019). The availability of general prenatal care has been shown to minimize the adverse effects of floods on pregnant women, as demonstrated in a study in Canada (Hetherington et al., 2021).

The study's findings revealed a lack of suitable places to care for the mother and baby after delivery. Like other flood‐affected individuals, the mothers lived in tents or inadequate housing with poor sanitation facilities. This finding is consistent with a study conducted in Japan, which found that women who gave birth in settlements faced numerous challenges in caring for their children, and living in temporary shelters presented various difficulties (Suzuki et al., 2022). However, there are discrepancies between the present study and the Nursal and Halawa (2021) study, which reported the availability of special support facilities and infrastructure for reproductive health programmes in the event of a disaster in the Sijunjung area. The facilities included emergency and maternity boarding rooms, maternity and child rooms, and rooms for infants and toddlers (Nursal & Halawa, 2021). These differences could be attributed to variations in how reproductive health services are supervised and monitored during disasters. Unlike in Iran, Sijunjung Regency appears to follow guidelines for the Minimum Initial Service Package (PPAM) for reproductive health in emergency response situations.

The variation in disaster response strategies and preparedness in different regions and countries can indeed impact the availability and effectiveness of reproductive health services during emergencies like floods. Having appropriate and well‐monitored reproductive health facilities and services in place during disasters is crucial to ensuring the well‐being of women and their newborns in such critical situations. Standardized guidelines and best practices can enhance disaster preparedness and response in reproductive health. By adopting these guidelines, healthcare systems can better plan, coordinate, and allocate resources to address the unique challenges presented by disasters (Beek et al., 2021; Sohrabizadeh et al., 2018).

Breastfeeding is a crucial factor in ensuring the health and well‐being of babies, especially in critical situations like floods. The present study highlighted the importance of providing special attention to breastfeeding mothers and supporting them to continue breastfeeding during and after the disaster.

Research has consistently shown that suboptimum breastfeeding practices can negatively impact child health and mortality. In emergencies, breastfeeding can become even more challenging due to various factors such as traumatic experiences, high stress levels, and emotional problems that may sometimes interfere with breast milk production (De Brabandere et al., 2014). Supporting breastfeeding mothers in emergencies is vital to overcoming these challenges. The study in Malaysia demonstrated that mothers require strong support from family members, community members, volunteers, and trained breastfeeding peer counsellors to encourage exclusive breastfeeding (Sulaiman et al., 2016).

The study findings indicate that pregnant and lactating women faced nutritional challenges during the flood, with their special dietary needs not being adequately addressed or given enough attention. Lack of snacks and essential food groups such as fruits, vegetables, and milk and dairy products were among the nutritional problems reported by women in the study.

The World Health Organization (WHO) guidelines emphasize the importance of providing additional food for pregnant women as part of their ration, either at home or on‐site, during emergencies like floods. Additionally, for lactating women, a ration card for the baby should be issued immediately and kept by the mother to ensure adequate nutrition exclusively for the mother, particularly during the first 6 months of the child's life (WHO, 2004). The study's findings align with research from Bangladesh, which identified floods as a root cause of malnutrition in breastfeeding mothers. The decrease in milk supply observed in breastfeeding mothers during floods can be attributed to consuming fewer varieties and amounts of foods due to limited access to nutritious food sources during the disaster (Mallett & Etzel Ra Md, 2018).

The present study's findings highlight the challenges faced in preparing infant formula during the flood and significant concerns for providing supplementary nutrition to infants and essential food for young children. These difficulties align with the findings of a study by Sulaiman et al. in Kelantan, Malaysia, which also identified infant feeding concerns during a flood. Infants are particularly vulnerable to health risks such as infectious diseases, malnutrition, and even death during floods. The reliance on donated infant formula during floods can also introduce challenges in ensuring proper and safe utilization, especially when access to clean water is compromised. It can hinder the safe preparation of formula, leading to non‐hygienic conditions that may put infants at risk of further health complications (Sulaiman et al., 2016).

The present study's findings align with previous research, indicating that the unscheduled distribution of baby formula during disasters can increase the risk of formula abuse. Easy access and unplanned distribution of baby formula in disaster‐affected areas can lead to substituting breast milk with formula feeding (Mudiyanselage et al., 2022) and undermining women's breastfeeding efforts (Nutrition Wing Ministry of National Health Services, 2017).

The present study's findings emphasize the importance of forecasting and preparing contraceptive supplies in the early stages of a flood or disaster response to meet the reproductive health needs of women. Lack of availability and utilization of contraceptives might pose the risk of increased unwanted pregnancies and shorter intervals between deliveries, as indicated in previous research (Loewen et al., 2021).

The findings of the present study underscore the challenges faced by women affected by the flood regarding menstrual hygiene. The lack of access to sanitary pads, blood‐spotted clothes, embarrassment, delayed replacement of menstrual pads due to unsuitable conditions, and the need for painkillers and iron pills were all identified as significant issues during the flood.

These challenges are consistent with research on humanitarian emergencies, which reveals that girls and women often lack reliable access to safe, private, and functional toilets, bathing facilities, and water supplies. Without these essential facilities, women may resort to harmful practices such as limiting their food and fluid intake, wearing menstrual materials for extended periods, and neglecting personal and menstrual hygiene. The lack of proper hygiene facilities can also compel women to address their hygiene‐related health needs in unsafe locations, exacerbating health risks during disasters (UNFPA, 2021). Moreover, the fear and anxiety experienced by women due to blood stains on their clothes are well‐documented in previous studies (Soeiro et al., 2021).

The present study's findings highlight the specific needs and challenges faced by high‐risk and vulnerable women during floods, including addicted women, women with HIV and AIDS, and women with physical and mobility limitations. This finding aligns with the results of previous research, indicating that vulnerable populations, such as the elderly, minority groups, and substance abusers, require special attention and support during natural disasters (Benevolenza & DeRigne, 2019).

On the other hand, women with disabilities are particularly affected during floods, as they may face mobility limitations that hinder their ability to perform daily tasks independently. Additionally, they may have difficulty understanding early warning signs of disasters, making them more reliant on the support of others, including social workers (Matlakala et al., 2021). People with HIV and AIDS are also significantly impacted during floods, with limited access to antiretroviral drugs, poor hygiene, poor nutrition, and disease‐induced stigma, all contributing to the challenges they face during disasters (Anthonj et al., 2015).

The study also revealed that the quantity and quality of sexual intercourse diminished during floods due to various limitations, such as a lack of suitable places, crowded living conditions, the unavailability of sexual partners, and a lack of facilities for ablution after intercourse. This decline in sexual activity can affect individuals' moods and relationships. Similar findings were reported in previous studies, where stressful living conditions, fatigue, and lack of privacy due to shared tents or living in groups were associated with decreased sexual desire and activity during disaster situations (Kamaledini & Azkia, 2021; Kohan et al., 2016).

The study's findings highlight the significant impact of men's absence during floods on women's well‐being and responsibilities. The lack of male presence at home for extended periods can create challenges for women, such as difficulty cleaning and tidying up after the flood and caring for children alone. This finding aligns with the results of previous research, indicating that women often bear the brunt of household and family responsibilities during disasters. At the same time, men may have external responsibilities or be away from home for various reasons. Despite these challenges, being together with family members during disasters can also positively affect individuals' mental well‐being (Rouhanizadeh & Kermanshachi, 2020).

The study's findings highlight valid concerns about the security and safety of women and girls during floods, particularly in temporary settlements or camps. The presence of strangers in these camps and factors such as lack of adequate lighting and distance from health facilities can make women vulnerable to violent attacks by strangers. This finding aligns with the results of previous research, which have indicated that living in refugee camps or temporary settlements can expose women to various gender‐related concerns, including violence and harassment (Bradley et al., 2021).

Violence against pregnant women, whether from spouses or strangers, was also reported in the present study. Factors like addiction and stress related to the floods were identified as potential contributors to such violence. These findings are consistent with other studies that have demonstrated an increase in various forms of violence during natural disasters (Brabete et al., 2021). For instance, in Japan, the prevalence of physical intimate partner violence (IPV) against pregnant women in earthquake‐affected areas was significantly higher than the national average (Sakurai et al., 2017).

### 5.1 Limitations of the study

The study was conducted on women affected by the flood in Aq Qala Township in Golestan, who belong to the Turkmen ethnic minority in Iran, highlighting the need for caution when generalizing the results to other populations. Additionally, the interval between the floods and the interviews, which was 2 years, may have implications for the accuracy of the participants' recollections, and some experiences or details related to the floods may be forgotten or altered with time.

### 5.2 Recommendations for further research

This study provides valuable insights into the reproductive health challenges faced by women during floods. Expanding the scope of research to include other crises and disasters, such as earthquakes, storms, and other natural disasters, and conducting such studies within the shortest time interval possible from the incident can develop more comprehensive and adaptable guidelines for managing women's reproductive health during emergencies.

## 6 CONCLUSION

Meeting women's reproductive health needs during floods poses several challenges, particularly in prenatal, postpartum, and breastfeeding care. Non‐pregnant women also experience difficulties related to menstrual health and sexual issues during floods. Additionally, the study found that, as with most natural disasters, women are at risk of experiencing violence in various forms during floods due to the changing living environment and increased stress. This issue can lead to a loss of women's sense of security.

Based on the study's findings, it appears that to adequately address women's reproductive health needs during floods, enhancing access to information and providing comprehensive SRH services for the affected population are crucial. Additionally, MISP programmes should be seamlessly integrated into relevant agencies and departments' national disaster preparedness and emergency planning. Adequate capacity must be developed at various levels to facilitate its implementation. It is important to note that these findings are based on a limited number of participants and cannot be generalized to a larger population.

## FUNDING INFORMATION

There has been no significant financial support for this work that could have influenced its outcome.

## CONFLICT OF INTEREST STATEMENT

There was no conflict of interest in this study.

## Data Availability

The data that support the findings of this study are available from the corresponding author upon reasonable request.
